# Modality of fear cues affects acoustic startle potentiation but not heart-rate response in patients with dental phobia

**DOI:** 10.3389/fpsyg.2015.00170

**Published:** 2015-02-27

**Authors:** André Wannemüller, Gudrun Sartory, Karin Elsesser, Thomas Lohrmann, Hans P. Jöhren

**Affiliations:** ^1^Department of Clinical Psychology and Psychotherapy, Ruhr-Universität Bochum, Bochum, North Rhine-Westphalia, Germany; ^2^Department of Clinical Psychology and Psychotherapy, University of Wuppertal, Wuppertal, North Rhine-Westphalia, Germany; ^3^Dental Clinic, Augusta Hospital, Bochum, North Rhine-Westphalia, Germany

**Keywords:** acoustic fear cues, phobic heart rate response, dental phobia, acoustic startle response, fear potentiated startle response, sensory gating, sensorimotor gating

## Abstract

The acoustic startle response (SR) has consistently been shown to be enhanced by fear-arousing cross-modal background stimuli in phobics. Intra-modal fear-potentiation of acoustic SR was rarely investigated and generated inconsistent results. The present study compared the acoustic SR to phobia-related sounds with that to phobia-related pictures in 104 dental phobic patients and 22 controls. Acoustic background stimuli were dental treatment noises and birdsong and visual stimuli were dental treatment and neutral control pictures. Background stimuli were presented for 4 s, randomly followed by the administration of the startle stimulus. In addition to SR, heart-rate (HR) was recorded throughout the trials. Irrespective of their content, background pictures elicited greater SR than noises in both groups with a trend for phobic participants to show startle potentiation to phobia-related pictures but not noises. Unlike controls, phobics showed HR acceleration to both dental pictures and noises. HR acceleration of the phobia group was significantly positively correlated with SR in the noise condition only. The acoustic SR to phobia-related noises is likely to be inhibited by prolonged sensorimotor gating.

## INTRODUCTION

Dental phobia is a specific phobia with extensive cognitive, physiological and behavioral symptoms (Schmid-Leuz et al., 2007; Sartory et al., 2009). Dentists are reported to be reluctant to treat dental phobics with one of the reasons being their increased startle reactivity during dental treatment which increases the risk of accidents (Cooper et al., 1980).The startle reaction (SR) is an automatic reflexive response to sudden or unexpectedly intense stimulation (Landis and Hunt, 1939). A continuously growing body of literature has shown that the SR varies with the individual’s affective state (for a review see Dawson et al., 1997).

Enhancement of acoustic SR was consistently reported during exposure to threatening or unpleasant background materials whereas positively evaluated stimuli were often accompanied by inhibition of the acoustic SR (Cuthbert et al., 1996; Vanman et al., 1996). Bradley et al. (1990) tested the effects of startle magnitude change by different affective picture materials applying both visual and acoustic startle probes. The results indicated that, regardless of probe modality, startle modulation was determined by the emotional valence of the pictures. Some studies investigated the impact of modality-compound stimuli such as emotional film-clips (Jansen and Frijda, 1994; Kaviani et al., 1999) on the acoustic SR. Others presented the affect-inducing stimulus in other than the visual channel, for instance by applying pleasant or unpleasant odors (Miltner et al., 1994; Kaviani et al., 1998) or sounds instead (Bradley and Lang, 2000). The latter study utilized naturally occurring sounds (screams, baby laughter etc.) with a visually elicited SR. All studies reported affective modulation effects of SR which is why this phenomenon is widely considered as being robust and modality-independent. It is, however, noteworthy that, although most studies used acoustic startle probes, only few applied an intra-modal design with both startle and lead stimuli being presented in the acoustic modality. These studies showed partially inconsistent results. Whereas Roy et al. (2009) reported an effect of pleasant and unpleasant music on the acoustic SR, Lipp et al. (1997) failed to find an intra-modal affective modulation effect. No firm conclusions can therefore be drawn as to the intra-modal affective startle modulation in the acoustic domain at this stage.

Similarly inconsistent results were found in studies with clinical groups. In patients with social phobia (Larsen et al., 2002; McTeague et al., 2009), post traumatic stress disorder (Miller et al., 2004) and specific phobia (Globisch et al., 1999; Hamm et al., 1997) fear-potentiated SR was reported upon exposure to phobia-relevant or personalized trauma-related materials. But there are also discrepant findings (Elsesser et al., 2004; Mühlberger et al., 2006; Sartory et al., 2009). Animal phobics consistently showed fear potentiation of the SR (Hamm et al., 1997; Sabatinelli et al., 2001; Mühlberger et al., 2006), whereas blood injury phobics showed once a lack (Sarlo et al., 2010) and in another study the presence of a fear-potentiated SR (Hamm et al., 1997). Methodological differences between studies could explain the discrepant results, not least the age difference between samples. A lower magnitude of the auditory SR has been reported with increasing age (Ellwanger et al., 2003; Ludewig et al., 2003) conceivably reflecting age-specific changes of central processing in the brain-stem centers involved in startle generation.

The previous studies involving clinical groups used cross-modal stimulation—presenting pictures as background stimuli and an acoustic startle stimulus—with the exception of one study with an intra-modal design (Sartory et al., 2009) in dental phobics. The effect of dental treatment noises on an acoustic startle stimulus was compared to that on bird song. Although the former were rated more fear-arousing, they failed to induce a fear-potentiated SR, in some instances even inducing inhibition. Two competing explanations were advanced to explain the result. First, the result may be situation-specific. The startle response (SR) may have been inhibited due to a functionally learned “keep still” response in the dental chair, i.e., participants may have learned to remain motionless during dental treatment to avoid injuries. This would be indicative of a measure of adaptiveness of the SR. Second, the inhibition may have been modality-specific due to an extended form of intra-modal sensorimotor gating (Geyer and Braff, 1987). In the latter case, the processing of a second of two stimuli is prevented so as to protect the processing of the first one.

A direct comparison between similarly threatening cross- and intra-modal lead stimuli could decide in favor of either of the alternatives. The respective effects of fear-arousing stimuli of different modalities on the acoustic SR have so far not been directly compared. In the present study, we investigated the fear potentiating effect of the acoustic SR due to pictures and noises of dental treatment in dental phobics. Both lead stimulus types were compared to emotionally neutral ones. In addition to the SR, the evoked phasic heart-rate (HR) reaction to stimuli was assessed. Unlike non-phobics, specific phobic participants usually show an accelerative HR response to the presentation of phobia-relevant material (e.g., Sartory et al., 1987; Hamm et al., 1997; Globisch et al., 1999) and the extent of the acceleration has been found to be linearly related to subjectively rated fear (Sartory et al., 1977). HR can therefore serve as an indicator of the fear-arousing properties of the material independently of SR-modulation. In addition, a small group of non-phobic control participants was included to ascertain the previously reported group differences with regard to HR reactions.

If both types of fear-arousing lead stimuli inhibit SR in dental phobics, the effect is likely to be due to a learned and functional “keep still” response appropriate while receiving dental treatment. If, however, only the intra-modal condition results in inhibition of the SR, the result is conceivably due to prolonged sensorimotor gating.

## MATERIALS AND METHODS

### PARTICIPANTS

Participants were 104 dental phobia patients and 22 non-phobic controls. The patients were recruited at the Dental Anxiety Clinic of the Augusta Hospital Bochum and included in the study if they met DSM-IV criteria for specific (dental) phobia. A clinical psychologist assessed the patients with a structured interview [Diagnostisches Interview bei psychischen Störungen (DIPS); Schneider and Margraf, 2006, the German adaptation of the Anxiety Disorders Interview Schedule for DSM-IV; ADIS-IV, Brown et al., 1994] to confirm DSM-IV criteria of a specific dental phobia and determine comorbid disorders. The DIPS has a good test–retest reliability (*r* = 0.64–0.89) and inter-rater reliability (kappa *r* = 0.80–1.00; Schneider and Margraf, 2006). The following comorbid disorders were diagnosed: 27 additional specific phobias, nine other anxiety disorders, seven affective disorders, four substance abuse or dependence, one personality disorder, one hypochondria. The phobic patients avoided dental appointments for *M* = 9.55 (SD = 8.04) years. The control group reported not to suffer from dental fear and denied having any previous psychological treatment. The study was approved by the ethics committee of the University of Wuppertal. All participants gave their written informed consent before being included in the study. Patients received psychological treatment after taking part in the experiment.

### EXPERIMENTAL DESIGN

An acoustic and a visual stimulation block consisted of eight phobia-related and eight neutral randomly interleafed lead stimuli, respectively. Half of them (four neutral and four phobia- related stimuli) were randomly followed by a startle noise in both blocks. The order of presentation was balanced. Half of the dental phobia patients and healthy controls were exposed to the visual followed by the acoustic lead stimuli and vice versa in the other half. The stimulus presentation was controlled by *SuperLab Pro 2.0* (Cedrus Corporation^®^).

### STIMULI

The acoustic startle stimulus consisted of a burst of broadband 105-dB[A] white noise, presented binaurally via headphones for 50 ms with an instantaneous (maximally 0.8 ms) rise and fall time. It occurred instantaneously after the 4-s presentation of the lead stimuli. SOAs varied randomly between 14 to 19 s.

Colored pictures presenting eight dental treatment scenes were taken partly from the International Affective Picture System (No. 9582, 9584; IAPS; Lang et al., 2008) and partly from other sources such as textbooks of dental treatment. The eight emotionally neutral pictures were taken from the IAPS (No. 5534, 7002, 7009, 7080, 7090, 7100, 7140, 7235), mainly consisting of household items. The pictures were projected onto a screen with a beamer (NEC^®^MultiSync MT 830+, Tokyo, Japan) resulting in a picture size of 105 × 75 cm. The participants regarded them from a distance of 1.7 m. The projection area was of an even gray color between stimulus presentations.

Phobia-relevant acoustic stimuli comprised eight kinds of noise related to dental treatment (scratching noises caused by two dental probes during an examination, two kinds of noises generated during ultrasound-cleaning to remove tartar, two noises of round bur drill and of two kinds of high frequency turbine drills). Bergmann et al. (2010) reported frequency ranges between 6.57 and 13.15 kHz for turbine drill sounds. Acoustic control stimuli consisted of eight kinds of birdsong (siskin, dunnock, yellow hammer, tree sparrow, goldfinch, chaffinch, lapwing, and linnet). Kreutzer and Güttinger (1991) measured a spectral range from 2.27 to 6.38 kHz for the yellow hammer’s bird song and Nowicki and Marler (1988) reported that the fundamental frequencies of bird song are typically within a range of 3–5 kHz and peaks up to 11 kHz. Acoustic stimuli were presented binaurally for 4 s each with an intensity of 60 dB[A].

Within each modality block, stimuli were randomized with no more than two stimuli of the same type in succession.

### PSYCHOPHYSIOLOGICAL MEASURES AND DATA REDUCTION

Psychophysiological data were recorded with a BIOPAC amplifier system (med-NATIC, Germany).

#### Startle reaction

The eye blink was recorded from the m. orbicularis oculi. Two miniature electrodes were placed below the right eye. The electromyogram (EMG) was recorded with a sampling rate of 1000 Hz. The signal was filtered so as to retain the 10- to 500-Hz frequency range, rectified, and integrated using a time constant of 15 ms. The maximum EMG response was determined within 40–200 ms after onset of the startle stimulus and base-line corrected taking the mean 130 ms before stimulus onset into account. Startle was averaged as magnitude. We defined SR to be valid if they were at least twice as high as the baseline EMG and would have excluded subjects if more than one startle trial per category had been invalid. In case of one missing startle the missing data were replaced by the individual’s mean score of the particular category. Applying that standard no participant had to be excluded. Following the recommendations of Blumenthal et al. (2005) SR-magnitudes of every individual were T-standardized z × 10 + 50 to eliminate inter-individual differences in responding. Taking all 1872 recorded SRs into account, mean peak reaction time was 105.83 ms (SD = 36.31).

#### Heart-rate

The electrocardiogram was recorded using chest electrodes. The sampling rate was 512 Hz; R-waves were detected online and converted into interbeat intervals (bpm) with one RR-interval delay. Mean HR was calculated for six 1-s epochs after stimulus onset and baseline-corrected taking 1-s before stimulus onset into account. HR reactions were averaged within stimulus categories resulting in evoked responses to the phobia-related and neutral stimuli. Mean 1-min HR prior to the start of the experiment served as resting HR.

#### Respiration rate

Respiration rate was recorded with a respiratory belt placed around the chest. Recordings were inspected and employed for artifact control.

### QUESTIONNAIRES

#### Dental anxiety scale

Dental anxiety scale (DAS; Corah, 1969; German version translated by the authors). This self-rating questionnaire consists of four items relating to dental treatment. Scores range from 4 to 20. A mean score of 9.07 was reported in 2103 non-selected participants and a mean score of 17.20 (SD = 1.80) in dental phobics (Corah et al., 1978). The German version had in internal consistency of Cronbach’s α = 0.64 (Sartory et al., 2006).

#### State-trait anxiety inventory

State-trait anxiety inventory (STAI X1, X2; Spielberger et al., 1970, German version by Laux et al., 1981). Subjects are asked to indicate the degree to which a given statement applies to them at present (state) and during the last two weeks (trait version). Scores range from 20 (no anxiety) to 80 (high anxiety). An internal consistency score of Cronbach’s α > 0.90 was reported (Laux et al., 1981).

#### Beck depression inventory

Beck depression inventory (BDI; Beck et al., 1961, German version by Hautzinger et al., 1994). This 21-item inventory indexes depression severity with a score range of 0–63. The authors reported internal consistencies between Cronbach’s α > 0.74 in healthy subjects and 0.92 in depressed patients.

#### Mutilation questionnaire

Mutilation questionnaire (MQ, Klorman et al., 1974, German version by Sartory and Brandl, 1992) was used to assess the extent of blood injury phobia among patients.The MQ consists of 30 items (applies—does not apply) and has a range from 0 to 30.

### PROCEDURE

Dental patients meeting the DSM-IV criteria for specific dental phobia and consenting to the procedure completed the questionnaires and attended the laboratory experiment a week later. After attachment of the electrodes, the room lights were dimmed and the startle stimulus was administered three times for the sake of demonstration before the start of the data acquisition. After an initial 3-min rest period during which resting HR was recorded the 16 pictures were presented followed by the noises (or vice versa). Participants were informed of the change of modality after the first block. Afterward all pictures and tones were again administered and participants were asked to rate the emotional valence (1 = pleasant; 9 = unpleasant) and fear-eliciting properties (1 = not at all; 9 = very strong) of the stimuli on a keyboard.

### DATA ANALYSIS

Groups (phobics vs. controls) were compared with regard to demographic and clinical variables and ratings of the stimulus materials. SR and HR were submitted to ANOVAs comparing groups x modality (pictures vs. noises) × phobia relevance (phobia-related vs. neutral stimuli) with a repeated measurement design followed by within-group analyses. Finally, age, questionnaire data, years of avoidance of dental treatment were correlated with HR and SR within the phobia group. All statistical analyses were carried out with the IBM SPSS Statistics 20 program.

## RESULTS

### DEMOGRAPHIC AND CLINICAL DATA (Table 1)

**Table 1:** **Demographic and clinical data of dental phobic patients and controls**.

	**Phobics (*N* = 104)**				**Controls (*N* = 22)**			
			**%**	**N**			**%**	**N**
Sex								
men			34.55	40			18.20	4
women			65.45	64			81.80	18
**Variable**	**M**	**SD**	**Range**	**N**	**M**	**SD**	**Range**	**N**
Age (years)**	37.15	11.25	17–62	104	24.55	5.66	20–45	22
Education (years)**	10.90	1.36	8–13	104	12.86	0.64	10–13	22
*Clinical status*								
Dental fear								
DAS***	17.36	2.13	13–20	104	8.23	2.67	4–13	22
Depression (BDI)**	11.15	8.89	0–49	103	4.59	3.94	0–19	22
Fear (STAI-State)**	57.52	12.12	32–79	102	35.96	7.34	23–52	22
Anxiety (STAI-Trait)*	42.93	10.31	23–70	103	36.91	8.84	20–57	22
Mutilation fear (MQ)	14.38	6.34	2–28	84	12.00	5.60	2–22	22

Note. Group difference ***p < 0.001, **p < 0.01, *p < 0.05.

Group comparisons with *t*-test showed that patients were significantly older *t*(124) = 7.72 and attended school for fewer years, *t*(124) = 10.92 than controls. Patients reported significantly higher dental fear (DAS), *t*(124) = 17.46, state anxiety (STAI-State), *t*(122) = 10.89, trait anxiety (STAI trait), *t*(123) = 2.83 and depression ratings (BDI), *t*(123) = 5.40 than controls.

### HEART RATE (Figure 1)

Owing to their taking cardiovascular medication, 16 phobia patients had to be excluded from analysis. Another six phobic and one control participants were excluded from analysis due to equipment malfunction.

#### Resting heart rate

Prior to the experiment, the resting HR (bpm) of phobic patients, *M* = 77.31 (SD = 12.86) was significantly higher than that of controls, *M* = 70.55 (SD = 8.19), *F*(1,100) = 5.23, *p* < 0.05, η^2^ = 0.05. A group comparison of the pre-stimulus second across trials yielded no significant results.

#### Phasic heart rate

**FIGURE 1:**
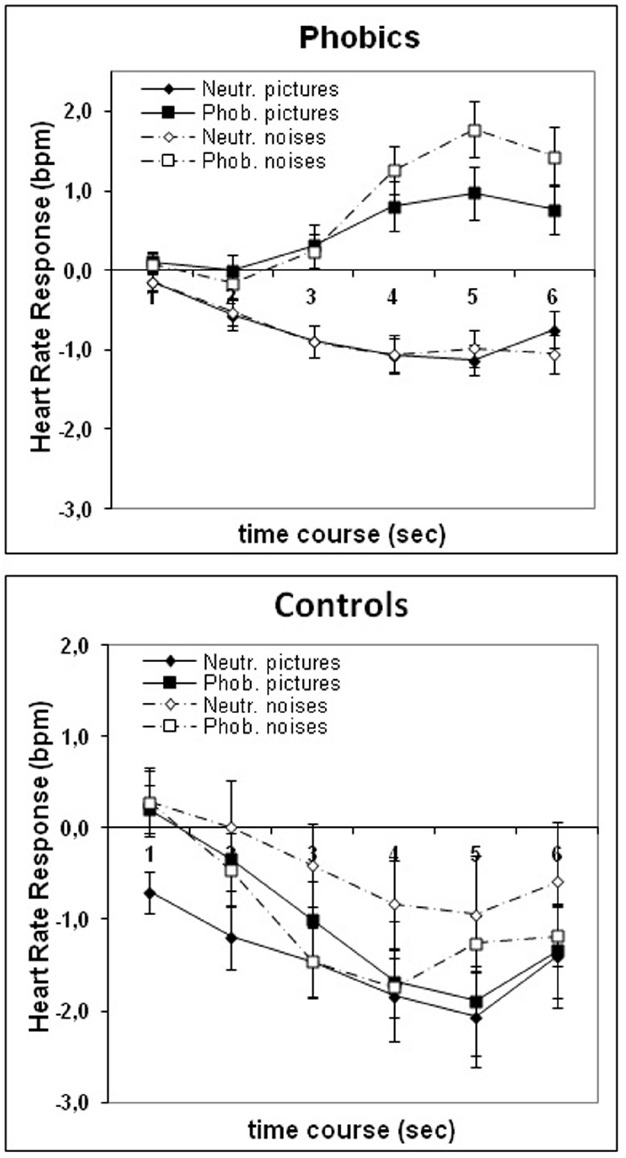
**Group means and standard errors of the evoked HR response to phobia-related and neutral pictures and noises in phobic and control participants**.

Groups were compared with regard to their evoked HR-responses to the stimulus categories with a 2 (group) × 2 (modality) × 2 (phobia relevance) × 6 (second) ANOVA with a repeated measures design. The analysis yielded significant main effects for group *F*(1,99) = 14.32, *p* < 0.001, η^2^ = 0.13, phobia relevance *F*(1,99) = 8.94, *p* < 0.01, η^2^ = 0.08 and second *F*(5,495) = 8.22, *p* < 0.001, η^2^ = 0.08. There were also significant interaction effects between group × second *F*(5,495) = 12.98, *p* < 0.0001, η^2^ = 0.12, group × phobia relevance *F*(1,99) = 10.70, *p* = 0.001, η^2^ = 0.10, and group × phobia relevance x second, *F*(5,495) = 11.10, *p* < 0.0001, η^2^ = 0.10. As shown in Figure 1 dental phobics reacted with HR acceleration to phobia-related and deceleration to neutral material whereas controls reacted to both types of stimuli with HR-deceleration.

Separate within-group analyses in the phobic group showed a significant effect for phobia relevance, *F*(1,79) = 44.97, *p* < 0.0001, η^2^ = 0.36, second, *F*(5,359) = 5.21, *p* < 0.0001, η^2^ = 0.06, phobia relevance × second: *F*(5,395) = 31.74, *p* < 0.0001, η^2^ = 0.29, modality × second *F*(5,395) = 2.19, *p* = 0.05, η^2^ = 0.03 and modality × phobia relevance × second, *F*(5,395) = 2.73, *p* < 0.02, η^2^ = 0.03. As shown in Figure 1, compared to neutral stimuli that elicited HR deceleration, phobia-relevant stimuli elicited HR acceleration with phobia-related noises evoking a higher response than phobia-related pictures in phobics. Within the control group there was a significant effect for second *F*(5,100) = 10.68, *p* < 0.0001, η^2^ = 0.35, indicative of the HR-deceleration seen in Figure 1.

### STARTLE RESPONSE (Figure 2)

**FIGURE 2:**
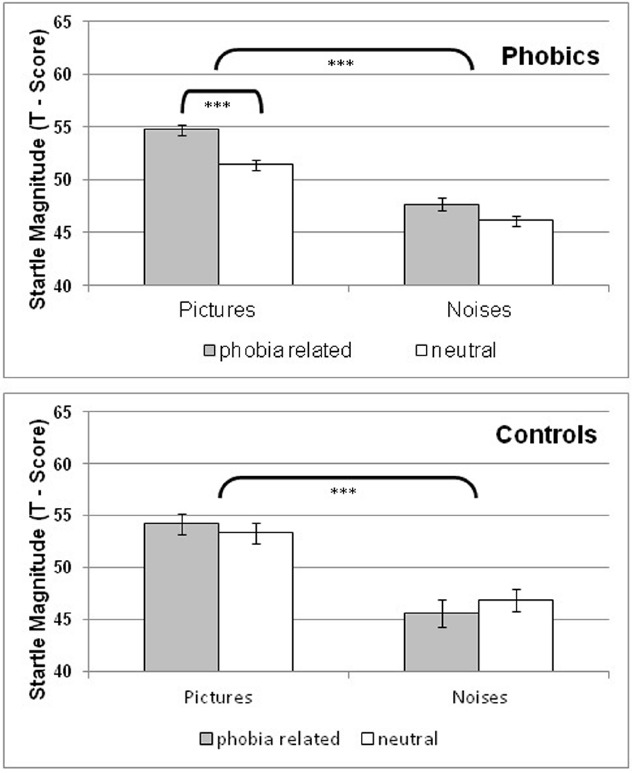
**Group means and standard errors of the startle response magnitude (EMG T-scores) following phobia-related and neutral pictures and noises in phobic and control participants**.

Owing to equipment malfunction 17 phobic and two control participants were excluded from SR analysis. SR was averaged across trials and submitted to ANOVA with a 2 (group) × 2 (phobia relevance) × 2 (modality) repeated measures design. There was a significant effect for modality *F*(1,106) = 61.65, *p* < 0.0001, η^2^ = 0.37, indicative of a larger cross than intra-modal SR-magnitude in both phobics and controls. Furthermore, there was a marginally significant interaction effect of group x phobia relevance, *F*(1,106) = 3.67, *p* = 0.06, η^2^ = 0.03. *Post hoc* analyses conducted in each group separately showed in phobics a significant effect for phobia relevance, *F*(1,87) = 16.42, *p* < 0.0001, η^2^ = 0.16, with phobia-related materials resulting in higher SR magnitudes than neutral materials, see Figure 2. Furthermore there was a marginally significant modality × phobia relevance interaction effect *F*(1,87) = 3.76, *p* = 0.06, η^2^ = 0.04. Among pictures SR enhancement to phobia-related material was highly significant *F*(1,87) = 28.21, *p* < 0.0001, η^2^ = 0.25, unlike the comparison between noises (*p* > 0.10). There was no significant phobia relevance effect among controls. Finally, a further modality × phobia relevance analysis was carried out in dental phobics after exclusion of all patients with comorbid disorders. The remaining 58 dental phobics yielded similar results with a significant modality (*p* < 0.001) and phobia relevance effect. There was also a significant phobia relevance effect for pictures (*p* < 0.0001) but none for noises.

When excluding all phobic participants with comorbid disorders (including those with other specific phobias) 58 subjects remained. Conducting a 2 (modality) × 2 (valence) ANOVA with repeated measurement correction at the omnibus level resulted in a highly sign. effect for modality [*F*(1,57) = 46,66, *p* < 0.0001, η^2^ = 0.45], and phobia relevance [*F*(1,57) = 13.80, *p* < 0.001, η^2^ = 0.20]. The modality × phobia relevance interaction was not significant (*p* = 0.11). Separate analyses of the two modalities yielded no significant effect for phobia relevance with regard to noise stimuli [*F*(1,57) = 2.29, *p* = 0.13, η^2^ = 0.04] and a highly significant one with regard to pictures [*F*(1,57) = 21.71, *p* < 0.0001, η^2^ = 0.28].

### SUBJECTIVE RATINGS (Figure 3)

**FIGURE 3:**
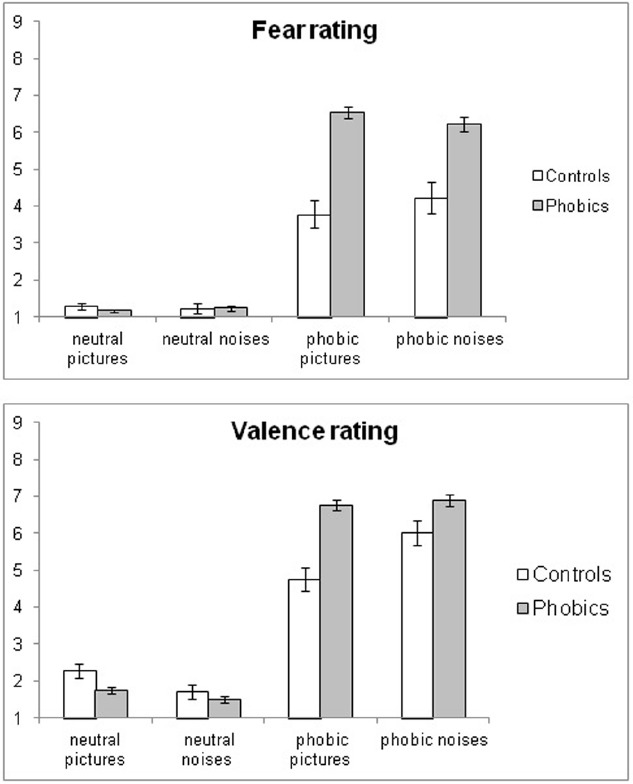
**Group means and standard errors of fear and valence ratings of phobia-related and neutral pictures and noises in phobic and control participants**.

Subjective fear and valence ratings were submitted separately to 2 (group) × 2 (modality) × 2 (phobia relevance) ANOVAs.

#### Fear ratings

There was a significant main effect for phobia relevance *F*(1,114) = 406.02, *p* < 0.0001, η^2^ = 0.78, and group *F*(1,114) = 26.28, *p* < 0.0001, η^2^ = 0.19. Significant interaction effects existed for group × phobia relevance *F*(1,114) = 38.28, *p* < 0.0001, η^2^ = 0.25 and group × modality x phobia relevance *F*(1,114) = 5.87, *p* < 0.02, η^2^ = 0.05.

In phobics, there was a significant effect for phobia relevance, *F*(1,95) = 1060.50, *p* < 0.0001, η^2^ = 0.92 and a significant interaction effect of modality × phobia relevance, *F*(1,95) = 6.60, *p* < 0.02, η^2^ = 0.07. The latter was due to phobia-related pictures, *F*(1,95) = 5.41, *p* < 0.03, η^2^ = 0.05, evoking higher fear ratings than noises.

The control group showed a significant effect for phobia relevance *F*(1,19) = 46.82, *p* < 0.0001 only, with phobic stimuli being considered more fear-inducing than neutral ones.

#### Valence ratings

The analysis yielded a significant main effect for group *F*(1,114) = 7.13, *p* < 0.01, η^2^ = 0.06, phobia relevance *F*(1,114) = 693.08, *p* < 0.0001, η^2^ = 0.86, group × phobia relevance *F*(1,114) = 30.77, *p* < 30.77, η^2^ = 0.21, group × modality *F*(1,114) = 4.74, *p* = 0.03, η^2^ = 0.04., modality × phobia relevance *F*(1,114) = 32.38, *p* < 0.0001, η^2^ = 0.22 and group × modality × phobia relevance *F*(1,114) = 13.77, *p* < 0.0001, η^2^ = 0.11.

In the phobia group there was a significant effect for phobia relevance *F*(1,95) = 1558.40, *p* < 0.0001, η^2^ = 0.94 with the phobia-related material being rated as more unpleasant than the neutral material. In controls there was also a significant main effect for phobia relevance *F*(1,19) = 102.40, *p* < 0.0001, η^2^ = 0.84, and a significant interaction effect of modality × phobia relevance *F*(1,19) = 25.93, *p* < 0.0001, η^2^ = 0.58. Phobia-related noises were rated more unpleasant than pictures, *F*(1,19) = 20.55, *p* < 0.0001, with an opposite trend in regard to the neutral material.

### CORRELATIONS BETWEEN MEASURES IN THE PHOBIA GROUP

Bivariate correlational analyses were carried out within the phobic sample only. Analysis involving HR was carried out only in those phobics who were not taking cardiovascular drugs. SR potentiation assessed by the difference between SR to phobia related and neutral stimuli and peak HR-response at the 5 s were correlated with questionnaire data and stimulus ratings as well as with years of avoiding dental treatment.

**FIGURE 4:**
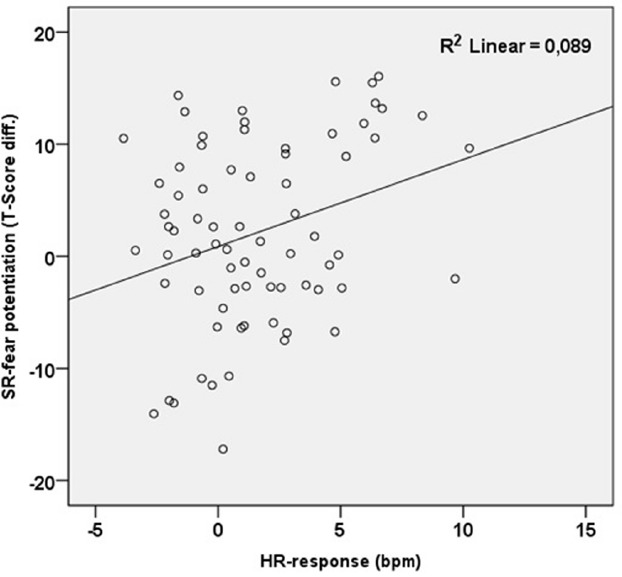
**Scattergram of HR response and intra-modal potentiation of SR-magnitude (phobia- related minus neutral) to dental noises**.

With regard to phobia-related noises, peak HR was significantly correlated with fear, *r* = 0.42, *p* < 0.0001, *n* = 78, and valence ratings, *r* = 0.30, *p* < 0.01, *n* = 78, as well as with SR potentiation, *r* = 0.30, *p* < 0.01, *n* = 73 (Figure 4). Peak HR to phobia-related pictures was significantly correlated with STAI state anxiety, *r* = 0.34, *p* < 0.002, *n* = 78. DAS-score was significantly correlated with fear- and valence ratings of phobia related pictures and noises (all *p* < 0.0001). Age, avoidance of dental treatment or DAS were not significantly correlated with psychophysiological measures.

## DISCUSSION

Both the phobic and control group showed greater SRs to cross- than intra-modal lead stimuli, i.e., to pictures than noises. The phobia group showed HR acceleration to phobia-related stimuli and HR deceleration to neutral stimuli with controls exhibiting HR deceleration to both types of stimuli. Although dental phobics rated phobia-related pictures as more fear-inducing than noises, the latter evoked greater HR acceleration than the former. However, there was a trend for phobia-related pictures but not noises to result in SR potentiation in the phobic group. HR acceleration was significantly correlated with SR potentiation in case of phobia-related noises only. Controls showed no SR potentiation to phobia-related material.

The HR response to phobia-related stimuli clearly differed between phobic and control participants with the former showing an accelerative response and the latter a decelerative one while both groups showed HR deceleration to neutral material. This finding has been reported repeatedly in specific phobics (e.g., Sartory et al., 1982, 1987, 2009; Globisch et al., 1999) as well as in patients with posttraumatic stress disorder to trauma-related stimuli (Elsesser et al., 2004, 2005). HR acceleration to the phobia-related stimuli is thought to be indicative of the presence of a fear network combining situational cues with neuroendocrine responses. HR deceleration has been associated with an interest or orienting reaction (Spinks and Siddle, 1983). It can therefore be concluded that only the phobic participants reacted with activation of a fear network to dental stimuli whereas control participants reacted with an attentional response to all stimuli alike.

Irrespective of the pattern of the HR response, SR were greater to pictures than to noises. Both groups showed decreased SR magnitude to acoustic compared to visual lead stimuli. Three factors have been suggested to diminish the acoustic SR namely, sensory masking (Campeau and Davis, 1992), the acoustic reflex (Silman, 1984) and sensorimotor gating (Geyer and Braff, 1987) related to prepulse inhibition (PPI). Sensory masking can be ruled out as the lead stimuli and startle probes were administered consecutively in the present study. The acoustic reflex protects the inner ear against harmful noise energies but was reported not to be evident with sound intensities of less than 70 dB (Silman, 1984). In the present study, the sound level of the background noises was 60 dB which appears to rule out the occurrence of the acoustic reflex.

Sensori-motor gating or (PPI; Braff and Geyer, 1990) is considered the system time required for encoding a first stimulus thereby preventing the processing of a second one. In contrast to findings with cross-modal stimulation, our results indicate that the prolonged sensory gating due to intra-modal stimulation interfered with the SR fear potentiation. The result corroborated the previous finding by Sartory et al. (2009) of a lack of fear-potentiated startle to auditory dental lead stimuli compared to neutral birdsong in dental phobics. Studies examining sensori-motor gating employed intra-modal stimuli throughout. They have, however typically used SOAs of 500 ms and less rather than the 4000 ms of the present study. It is conceivable that due to their higher complexity, noises may also have required more time and processing resources for their identification with regard to their content than the simple stimuli of the previous studies. It could also be speculated that the complexity of the noise stimuli compared to the constant visual stimulation pattern presented by the pictures accounted for the present difference in results of SR-potentiation. However, patterned lead stimuli have previously been reported to result in affective SR-modulation with highly complex stimuli such as filmclips (Jansen and Frijda, 1994; Kaviani et al., 1999), imagery (e.g., Cook et al., 1991; Hawk et al., 1992), or music (Roy et al., 2009). Further research using intra-modal designs appears necessary to investigate the effect on affective SR-modulation of complexity and duration of the intra-modal lead stimulus.

There was a trend for phobic participants to show acoustic SR potentiation only to dental pictures but not to dental noises. The result must be viewed with some caution as the preceding overall analysis was not significant with regard to the modality x phobia relevance effect and the subsequent within-group analysis only marginally so in the phobia group. This significant interaction may be partly due to the apparently (although not significantly) higher SR to neutral pictures in controls than phobics. However, the significant SR potentiation to pictures in phobics compared to controls could also be due to the former’s greater discrimination between the two types of stimuli. Finally, it cannot be ruled out that phobics showed SR potentiation to pictorial but not acoustic stimuli because they experienced them as being more fear-evoking as shown in their fear ratings.

The correlations between measures confirm the previous finding of an association between subjective fear and HR acceleration to phobia-related stimuli in phobics (e.g., Sartory et al., 1987). Avoidance, in the present study defined as years since last dental treatment, has similarly not been found to be correlated with measures of self-report or HR acceleration in specific phobia (Sartory et al., 1982, 1990). It has to be concluded that factors additional to subjective fear regulate approach to the phobic situation. In the present study, SR potentiation was found to be related to HR acceleration in phobics in the acoustic lead stimulus condition. Reflex potentiation and sympathetic activation due to emotional reactions have been reported to be relayed via different projections from the amygdala (LeDoux, 1995), the former being effected via the nucleus reticularis pontis caudalis (Rosen et al., 1991) and the latter via the lateral hypothalamus and the rostral ventral nucleus of the medulla. It is likely that both efferents are similarly subject to increased activation. It is, however, noteworthy that the correlation proved significant only in the acoustic lead stimulus condition which, compared to the cross-modal condition, was characterized by SR inhibition but an increased HR reaction. If SR inhibition is indeed due to prolonged sensorimotor gating, it is conceivable that the high HR response interfered with the processing of the lead stimulus therefore reducing the sensorimotor gating effect. High phasic HR reactions have been found to be followed by a secondary long latency HR response in phobics (Sartory, 1986; Sartory et al., 1987). It is thought that they result from stress hormone release additional to the initial sympathetic activation. It could be argued that the increased level of stress hormones may also have interfered with processing of the complex acoustic lead stimuli.

Among the limitations of this study is the lack of a generally aversive control condition which could have given information as to whether the generally higher fearfulness of the patients or their specific dental phobia accounted for the increased SR to the unpleasant dental pictures. Furthermore, no startle probes were delivered during the inter-trial intervals. It could thus not be evaluated whether the neutral stimuli had an effect on SR and also not whether the intra-modal exposure to any auditory lead stimulus generally led to a sensitisation of SR. Additionally, in order to shorten the experimental procedure, we did not gather ratings of arousal and therefore cannot be certain whether this factor, or the lack thereof, may have failed to mediate the process of affect-modulated SR (c.f. Cuthbert et al., 1996), especially in controls. Finally, the small size of the control sample limits generalizations to be drawn from their results.

## AUTHOR CONTRIBUTIONS

Conceived and designed the experiment: GS, AW, KE. Analyzed the data: AW, TL. Contributed reagents/materials/analysis tools: HPJ, TL. Wrote the paper: AW, GS, KE.

### Conflict of Interest Statement

The authors declare that the research was conducted in the absence of any commercial or financial relationships that could be construed as a potential conflict of interest.
